# Comparative Expert Evaluation of Multimodal Large Language Models for Pediatric Rash Diagnosis: Clinical Utility, Safety, Information Quality, and Readability

**DOI:** 10.3390/children13091272

**Published:** 2026-09-18

**Authors:** Dilara Lahut, Özlem Erdede, Rabia Gönül Sezer Yamanel

**Affiliations:** Department of Pediatrics, Zeynep Kamil Women and Children’s Diseases Training and Research Hospital, University of Health Sciences, 34668 Istanbul, Türkiye; ozlem@erdede.com.tr (Ö.E.); rabiagonul@hotmail.com (R.G.S.Y.)

**Keywords:** pediatric dermatology, pediatric rash, large language models, artificial intelligence, ChatGPT, Gemini, Grok, clinical decision support, diagnostic accuracy, readability

## Abstract

**Highlights:**

**What are the main findings?**

- Multimodal LLMs generated predominantly clinically acceptable responses to pediatric rash vignettes, although performance varied substantially across clinical presentations.
- No consistent platform advantage was demonstrated after adjusted comparisons, and all three models failed to identify the reference diagnosis in at least one challenging vignette.

**What is the implication of the main finding?**

- Multimodal LLMs may support differential diagnosis and clinical review in pediatric rash assessment, but their outputs require clinician verification.
- Their role should be considered adjunctive rather than autonomous, particularly for uncommon or diagnostically challenging presentations.

**Abstract:**

**Background/Objectives:** Multimodal large language models (LLMs) can interpret clinical text and images, but their performance in pediatric rash assessment remains uncertain. This study compared the clinical utility, safety, information quality, diagnostic correctness, and readability of ChatGPT, Gemini and Grok. **Methods:** Fifteen content-validated pediatric rash vignettes with brief histories and anonymized photographs were submitted once to each platform using a standardized zero-shot prompt. Three pediatricians blinded to platform identity independently rated the 45 responses using a five-point Clinical Utility and Safety (CUS) scale and a five-item modified DISCERN instrument. Diagnostic correctness was assessed descriptively; platform comparisons used Friedman tests with Bonferroni-adjusted Wilcoxon tests when appropriate. **Results:** Overall, 82.2% of CUS ratings were in categories 4–5 and 83.0% of modified DISCERN scores were ≥20/25; no rating was assigned to CUS category 1. Gemini and Grok had descriptively higher expert ratings than ChatGPT, but CUS did not differ significantly across platforms (*p* = 0.157), and although modified DISCERN differed globally (*p* = 0.038), no pairwise comparison remained significant after adjustment. In the single-query diagnostic assessment, at least one platform missed the reference diagnosis in 9/15 vignettes, and all three missed porphyria. Gemini generated the longest responses, whereas Grok produced the most linguistically complex text; neither response length nor readability was associated with expert ratings. **Conclusions:** The three multimodal LLMs produced predominantly clinically acceptable responses, but performance varied by vignette and platform. Because each vignette–platform combination was sampled once, diagnostic findings represent single-response observations rather than stable platform accuracy estimates. Clinical verification remains necessary.

## 1. Introduction

Rash presentations are common in pediatric emergency departments and outpatient clinics, but their evaluation can be diagnostically challenging. The underlying causes are highly heterogeneous and include viral and bacterial infections, inflammatory and autoimmune disorders, hematologic conditions, drug reactions, arthropod-related eruptions, and inherited or metabolic diseases. Clinical features may also evolve over time, and different conditions can share similar lesion morphology and distribution, particularly during early or atypical presentations. Consequently, diagnostic errors may result in delayed recognition of serious disease, unnecessary investigations for benign conditions, or inappropriate treatment. Previous reviews have therefore emphasized the importance of structured clinical reasoning that integrates lesion characteristics, systemic findings, epidemiologic context, and warning signs [1,2,3].

Large language models (LLMs) are increasingly being explored as clinical support tools for documentation, triage, diagnostic suggestion, management planning, and health communication. Studies in emergency medicine have shown that contemporary models can achieve encouraging performance in selected tasks, including acuity classification and diagnostic ranking, and may approach physician-level accuracy in some structured adult scenarios [4,5,6]. More recent evaluations in pediatric emergency care have also reported promising but variable diagnostic performance, with substantial differences between models and clinical cases [7,8]. However, these systems may generate unsupported information, misinterpret clinical context, omit important safety considerations, or provide recommendations that conflict with established guidance [9]. In pediatric rash presentations, such errors may be particularly consequential because visually similar eruptions can represent conditions ranging from self-limited disease to time-sensitive infection, vasculitis, or severe drug reactions. Age-specific clinical context is also important, as dermatologic presentation and differential diagnosis may vary across age groups, and AI systems developed predominantly in adult populations may not generalize directly to pediatric patients [10].

Dermatology has been an active area of artificial intelligence research, particularly in image classification and the development of specialized systems trained on large collections of skin photographs [11,12,13]. Nevertheless, many such studies have focused on adult populations, isolated images, or task-specific algorithms rather than general-purpose conversational models that integrate clinical history with photographs. Pediatric rash assessment also requires integration of dermatologic morphology with age-specific and systemic clinical information, making it a relevant setting for evaluating multimodal LLMs. A recent large comparative study evaluated three AI models against 263 pediatric residents and specialists using 61 image-supported, multiple-choice childhood exanthem cases. ChatGPT and Gemini achieved diagnostic scores above the upper bound of the specialist population median, although performance varied considerably by disease and was lowest for conditions requiring greater contextual reasoning [14]. This study provides important evidence regarding diagnostic accuracy, but its multiple-choice design primarily assessed selection of a single diagnosis. In routine clinical use, however, pediatricians require more than a diagnostic label: AI-generated responses may also influence the construction of differential diagnoses, immediate management, recognition of warning signs, and decisions regarding further evaluation or specialist referral.

Accordingly, there remains a need to evaluate the broader clinical quality of open-ended multimodal LLM responses in pediatric rash presentations. In the present study, we compared three widely accessible general-purpose LLMs using standardized vignettes that combined clinical histories with lesion photographs. The primary outcomes were expert-rated Clinical Utility and Safety and modified DISCERN scores, reflecting the safety, reliability, completeness, and clinical appropriateness of the generated responses. Secondary outcomes included the correctness of the most likely diagnosis, response length, and readability characteristics. By assessing both diagnostic performance and the quality of accompanying clinical guidance, this study aimed to determine the potential and limitations of multimodal LLMs as supervised decision-support and educational tools in pediatric practice.

## 2. Materials and Methods

### 2.1. Study Aim and Design

**Figure 1:**
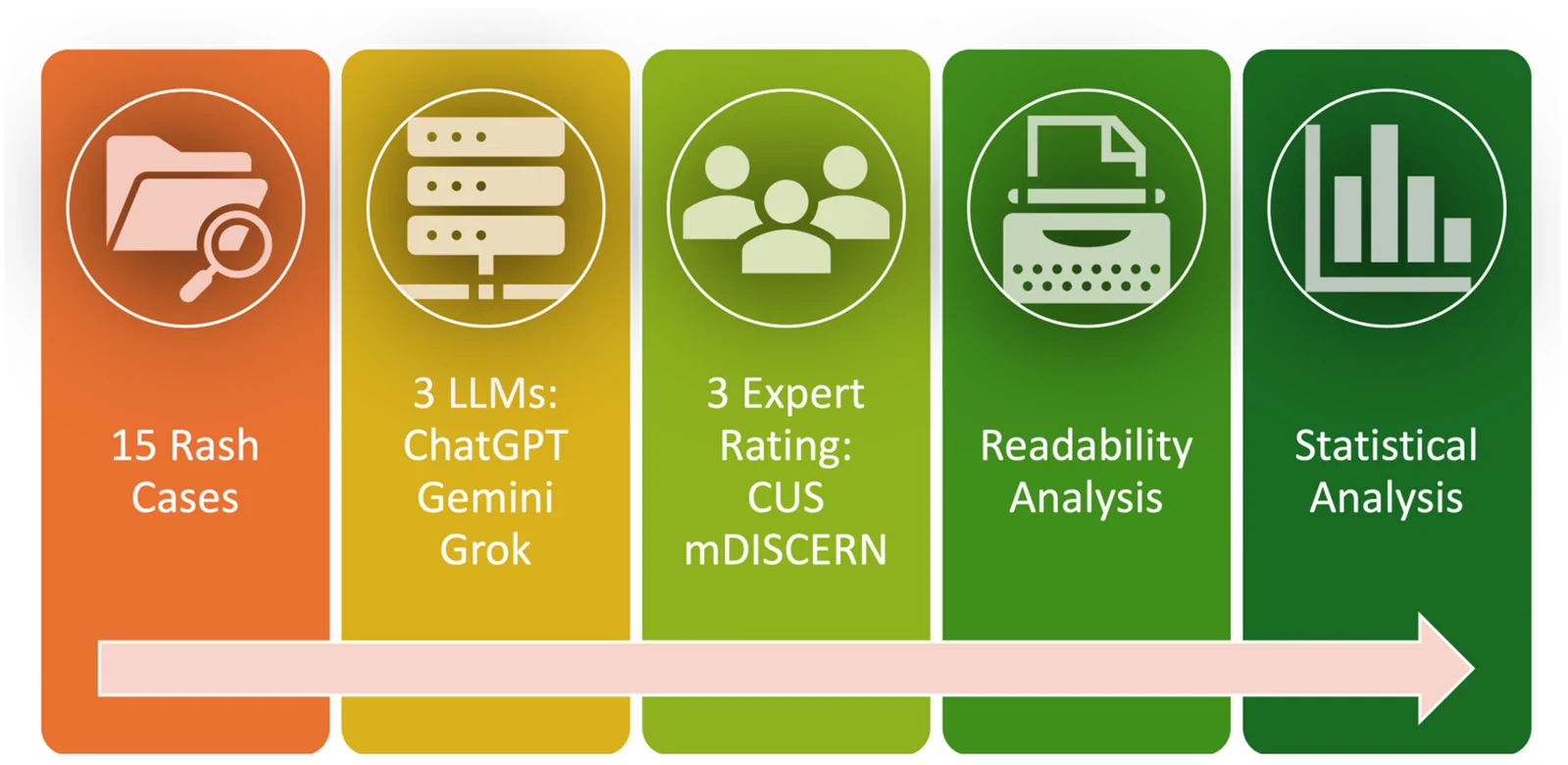
Study Design.

We conducted a comparative, vignette-based, cross-sectional evaluation of three general-purpose large language models (LLMs) in pediatric rash presentations. The primary aim was to compare the clinical utility, safety, and information quality of their open-ended diagnostic and management responses using expert-rated Clinical Utility and Safety (CUS) and modified DISCERN (mDISCERN) scores. Secondary outcomes included the correctness of the most likely diagnosis and the readability and text-length characteristics of the generated responses. Each platform was evaluated using its native multimodal vision capabilities, enabling simultaneous interpretation of the clinical vignette and accompanying lesion photograph(s). The study was conducted at a tertiary pediatric center between November and December 2025. The study workflow is presented in Figure 1.

### 2.2. Case Selection and Content Validation

The source material comprised historical clinical cases of children who had been admitted to the study hospital and had completed their diagnostic evaluation and treatment. Only cases with a clearly documented final diagnosis in the medical record were considered eligible. The reference diagnosis for each vignette was the final diagnosis documented after completion of the clinical diagnostic work-up, based on the overall clinical assessment and condition-specific investigations available during routine care, with specialist consultation when clinically indicated. Because the included conditions were heterogeneous, the diagnostic pathway and confirmatory investigations differed across vignettes, and no uniform confirmatory test was required.

Twenty candidate pediatric rash vignettes were initially prepared. Each vignette included a brief, structured clinical history covering the presenting complaint, relevant negative findings, and basic laboratory results, together with one or two representative clinical photographs of the skin lesions. Before use, all materials were fully de-identified by removing direct identifiers, masking recognizable facial features where necessary, and deleting image metadata.

To ensure broad and clinically relevant coverage, cases were purposively selected from infectious eruptions, immune-mediated and vasculitic disorders, drug- and arthropod-related eruptions, pigmentary disorders and genodermatoses, and nutritional or metabolic dermatoses.

Content validity was assessed using the Content Validity Index (CVI) framework [15,16]. Five independent volunteer pediatricians, each with at least 10 years of clinical experience and none of whom participated in the subsequent assessment of the LLM outputs, evaluated each vignette on a four-point scale from 1 to 4 for relevance, clarity, and representativeness of real-world pediatric rash presentations. For each assessed domain, the item-level CVI (I-CVI) was calculated as the proportion of experts assigning a rating of 3 or 4. Given the five-member expert panel, an I-CVI threshold of ≥0.80, corresponding to agreement by at least four of five experts, was used as the prespecified pragmatic retention criterion. Vignettes not meeting this threshold were excluded.

Five vignettes did not meet the prespecified I-CVI threshold, primarily because the experts considered them overly straightforward and insufficiently representative of the diagnostic challenge targeted by the study. These vignettes were excluded. The remaining 15 vignettes achieved an I-CVI ≥ 0.80 across all assessed domains and required no revision.

The final vignette set comprised erythema ab igne, eczema herpeticum, insect bite, ecthyma gangrenosum, IgA vasculitis (Henoch–Schönlein purpura), herpes zoster, acrodermatitis enteropathica, pustular psoriasis, variegate porphyria, juvenile dermatomyositis, papular acrodermatitis, café-au-lait macules, bullous impetigo, roseola infantum, and infectious mononucleosis. Full vignette texts and redacted clinical photographs are provided in Supplementary File S1.

### 2.3. LLM Platforms and Response Generation

Three widely accessible general-purpose multimodal LLM platforms were evaluated: ChatGPT (GPT-5.1; OpenAI, San Francisco, CA, USA), Gemini (Gemini 3; Google, Mountain View, CA, USA), and Grok (Grok 4.1; xAI, Palo Alto, CA, USA). All three platforms were accessed through their free consumer web interfaces, and the model versions reported here correspond to the model labels displayed by the respective interfaces on the query date. The platforms were selected because they were accessible to individual users through standard web interfaces, supported simultaneous processing of text and clinical images, and were capable of generating English-language clinical responses.

When a platform permitted access without user registration, it was accessed without an account. When account registration was required, a separate newly created account with no previous activity or conversation history was used for each vignette. Each vignette–platform interaction was initiated in a fresh session with no preceding conversation history. All platform settings were left at their default values to approximate routine user conditions. No optional reasoning or “thinking” mode, web-browsing or search function, or external tool was manually activated; only the standard multimodal chat interface and image-upload functionality were used.

A standardized zero-shot prompting strategy was used across all platforms. Each of the 15 vignettes was submitted once to each platform through its native web interface with multimodal vision capabilities enabled. All queries were completed on 9 December 2025 to minimize potential variability arising from interim model updates.

Each interaction began with the same fixed prompt:

“I am a pediatric resident. I will provide you with a pediatric case that includes a brief clinical history and a rash photograph. Please analyse the case as a pediatric clinician and answer under the headings: most likely diagnosis, ranked differential diagnoses, immediate management and safety considerations.”

The vignettes were submitted in the same order across all three platforms. For each vignette–platform combination, only the first complete response was retained. No regeneration requests, follow-up prompts, or manual modifications were permitted. The responses were exported as plain text, assigned anonymized platform labels (A, B, and C), and stored for subsequent expert assessment and statistical analysis.

### 2.4. Expert Evaluation and Outcome Measures

Each model response was evaluated across four domains: diagnostic appropriateness, clinical utility and safety, information quality, and linguistic characteristics. The primary outcomes were expert-rated CUS scores and mDISCERN total scores. Secondary outcomes included the correctness of the most likely diagnosis, readability indices, and basic text-length measures. The measures and their operational definitions are summarized in Table 1.

Clinical utility and safety were assessed using a study-specific five-point anchored CUS scale developed for the present evaluation. The scale assessed the overall clinical usefulness, safety, and concordance of each response with current pediatric practice, ranging from clinically dangerous or clearly incorrect advice to fully correct, safe, and clinically appropriate guidance. The exact CUS anchors provided to the raters are reproduced in Table 1.

Information quality was assessed using a five-item mDISCERN instrument adapted from the original DISCERN framework for clinician-facing guidance [17]. The adapted instrument evaluated scientific grounding, acknowledgment of alternatives and uncertainty, communication of risks, benefits, and contraindications, concordance with current pediatric standards, and the overall reliability and clarity of the response. Each item was scored from 1 to 5, yielding a total score ranging from 5 to 25. The exact item definitions and scoring framework provided to the raters are shown in Table 1.

For descriptive purposes, we additionally reported the proportion of ratings classified as clinically useful and safe (CUS categories 4–5) and the proportion with an mDISCERN total score of at least 20/25, corresponding to a mean item score of at least 4/5. These thresholds were used only to summarize score distributions and were not used to dichotomize outcomes in the main inferential analyses.

As a secondary diagnostic outcome, the diagnosis stated by each model as the most likely diagnosis was compared with the final diagnosis documented in the patient’s medical record. A response was classified as correct only when the stated most likely diagnosis matched the documented reference diagnosis or represented an unequivocal synonym. Broader or nonspecific diagnostic categories, clinically related but distinct conditions, and reference diagnoses mentioned only within the differential diagnosis were classified as incorrect. Diagnostic correctness was assessed separately at the vignette–platform level as a secondary descriptive outcome.

Readability and basic text structure were assessed for each response. The calculated readability indices were the Flesch Reading Ease Score (FRES), Flesch–Kincaid Grade Level (FKGL), Gunning Fog Index (GFI), Simple Measure of Gobbledygook (SMOG), and Coleman–Liau Index (CLI). Total word and sentence counts were also recorded as measures of response length. FRES is commonly interpreted on a scale from 0 to 100, with higher values indicating easier readability, whereas FKGL, GFI, SMOG, and CLI estimate the approximate educational level required to understand the text [18,19,20,21]. Because the responses were intended for clinicians rather than patients or the general public, no prespecified readability threshold was used to classify responses as adequate or inadequate.

Readability indices and text-length measures were calculated using Readable.com, a web-based calculator implementing established and widely used readability formulas [18,19,20,21,22].

Three experienced pediatricians who had not participated in vignette development or content validation served as expert raters. Before formal scoring, the raters completed a structured calibration exercise using five sample LLM responses that were not included in the analytical dataset. The purpose of the exercise was to establish a shared understanding of the CUS anchors and mDISCERN item definitions rather than to obtain consensus ratings.

Each rater subsequently evaluated all 45 model responses independently. The raters were blinded to the correspondence between the anonymized platform labels and the evaluated LLMs. However, they were members of the clinical team involved in the care and diagnostic evaluation of the source patients and were therefore aware of the documented reference diagnoses. Thus, the expert ratings represented reference-informed evaluations of the overall clinical responses rather than diagnosis-blinded assessments. No consensus meetings were held during or after formal scoring, and the original independent ratings were retained for analysis.

Ratings were based on current clinical evidence, standard pediatric references, relevant guidelines, and usual practice in a tertiary pediatric setting. Each response was evaluated as a whole, including the proposed most likely diagnosis, ranked differential diagnoses, immediate management recommendations, and safety considerations.

### 2.5. Statistical Analysis

The LLM platform was the main explanatory variable. CUS and mDISCERN scores were summarized at both the response and individual-rating levels. For continuous platform-level descriptive statistics, the three rater scores were averaged for each vignette–platform combination, yielding 15 response-level observations per platform; means and standard deviations were calculated from these response-level values. Individual ratings (45 per platform and 135 overall) were retained for describing score distributions and for reporting the proportions of ratings in CUS categories 4–5 and with mDISCERN total scores ≥ 20/25. Categorical score distributions were reported as counts and percentages.

Three platforms were compared separately for mean CUS and mean mDISCERN scores using the Friedman test. When the global Friedman test was statistically significant, pairwise comparisons were performed using two-sided Wilcoxon signed-rank tests. Because tied differences were expected, asymptotic *p* values were used. Pairwise *p* values were adjusted using the Bonferroni method.

Readability indices and text-length measures were calculated once for each of the 45 model responses. Because the same 15 vignettes were evaluated across all three platforms, platform differences in these outcomes were assessed using one-way repeated-measures analysis of variance, with vignette treated as the repeated-measures unit. When the global platform effect was statistically significant, paired comparisons were performed using paired-samples *t* tests. The resulting *p* values were adjusted using the Benjamini–Hochberg procedure separately for each readability or text-length outcome.

Diagnostic correctness was summarized descriptively as the number and percentage of vignettes for which each platform identified the documented reference diagnosis as its most likely diagnosis. No inferential comparison of diagnostic correctness was performed because only 15 vignettes were included and each vignette–platform classification was based on a single model response.

Exploratory associations between expert ratings and linguistic characteristics were examined at the vignette–platform level. Mean CUS and mDISCERN scores across the three raters were correlated with word count, sentence count, FRES, FKGL, GFI, CLI, and SMOG using two-sided Spearman rank correlations. Benjamini–Hochberg-adjusted *p* values were additionally calculated across the 14 exploratory correlations.

Inter-rater reliability for mDISCERN total scores was assessed across the 45 vignette–platform responses using a two-way random-effects intraclass correlation coefficient for absolute agreement. Both single-rater reliability, ICC(2,1), and reliability of the mean of three raters, ICC(2,3), were reported with 95% confidence intervals [23]. Agreement for the ordinal CUS ratings was assessed using Krippendorff’s alpha with an ordinal distance metric [24].

All tests were two-sided, and *p* values < 0.05 were considered statistically significant. Statistical analyses were performed using R software (version 4.5.1) and IBM SPSS Statistics (version 26). Results were cross-checked between the two software environments where applicable.

### 2.6. Ethical Considerations

The study was conducted in accordance with the Declaration of Helsinki and was approved by the Ethics Committee of Zeynep Kamil Women and Children’s Diseases Training and Research Hospital (approval no. 129; 22 October 2025). All vignette texts and clinical photographs were derived from historical cases that had completed diagnostic evaluation and treatment. Written informed consent for the use and publication of anonymized clinical information and accompanying photographs was obtained from the parents or legal guardians of all included patients. Before inclusion, all materials were fully de-identified by removing direct identifiers and image metadata and by masking recognizable facial features where necessary. The ethics committee-approved study protocol explicitly included the submission of de-identified clinical information and images to the evaluated third-party LLM platforms. Only fully de-identified materials were uploaded to these platforms. Participation of the pediatricians involved in content validation and expert rating was voluntary.

## 3. Results

### 3.1. Study Outputs and Overall Expert Ratings

The 15 pediatric rash vignettes generated 45 model responses across ChatGPT, Gemini, and Grok. Each response was independently evaluated by three pediatricians, resulting in 135 CUS ratings and 135 mDISCERN total scores, with no missing data.

**Figure 2:**
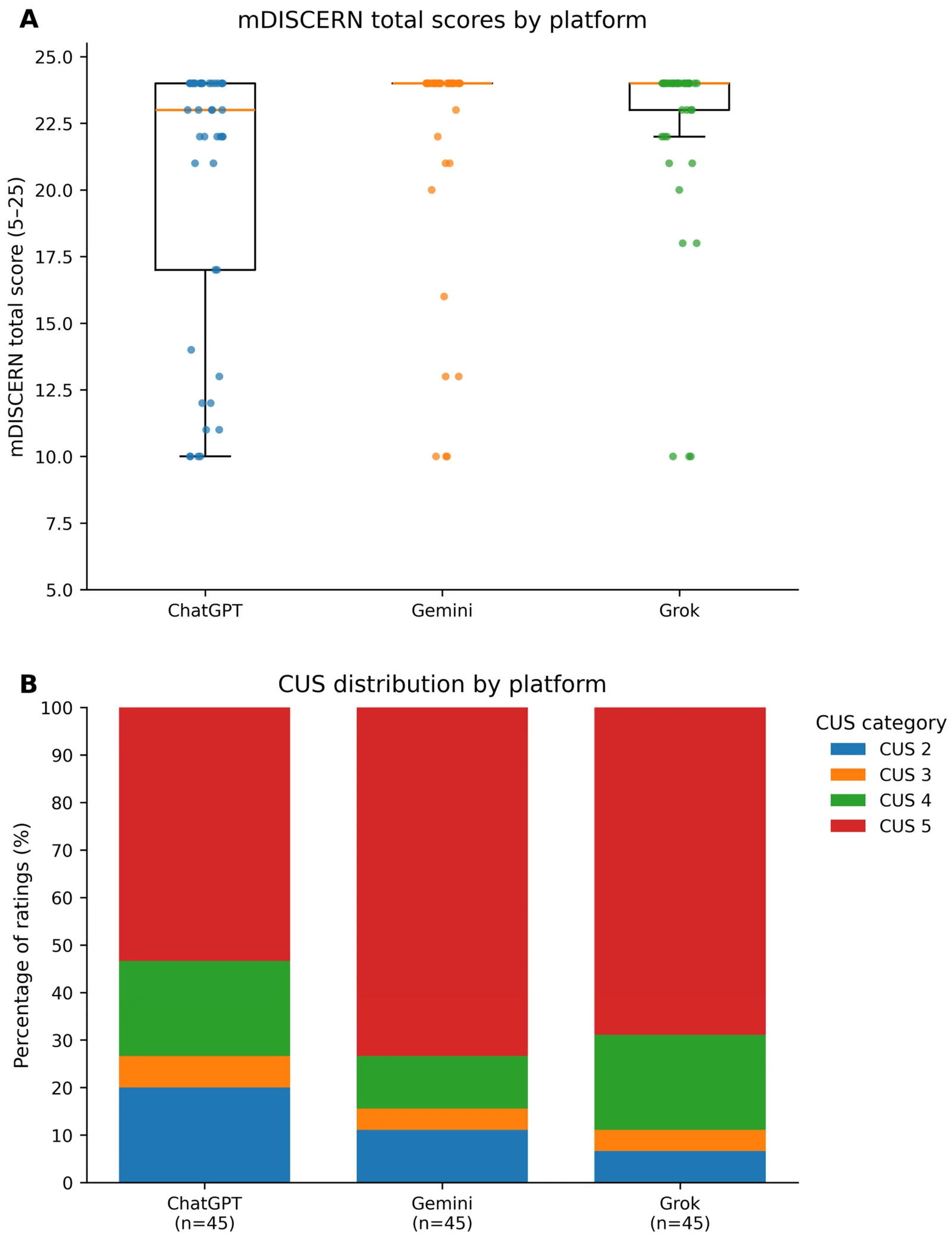
Expert-rated quality and safety outcomes by LLM platform. (**A**) Distribution of modified DISCERN total scores (mDISCERN; range: 5–25), shown as boxplots with individual rater-level observations overlaid. The horizontal line indicates the median, the boxes represent the interquartile range, and the whiskers extend to 1.5 times the interquartile range. (**B**) Percentage distribution of Clinical Utility and Safety (CUS) ratings by platform. Percentages were calculated using all ratings for each platform (*n* = 45). No rating was assigned to CUS category 1.

Overall, 111 of 135 CUS ratings (82.2%) were in categories 4–5, and no rating was assigned to CUS category 1. In addition, 112 of 135 mDISCERN total scores (83.0%) were ≥20 out of 25. Descriptive results by platform are presented in Table 2, and the distributions of expert-rated outcomes are shown in Figure 2.

Inter-rater reliability estimates for mDISCERN were high. The single-rater ICC was 0.964 (95% CI, 0.938–0.980), and the ICC for the mean of three raters was 0.988 (95% CI, 0.979–0.993). Ordinal agreement for CUS ratings was Krippendorff’s α = 0.743. Complete agreement among all three raters was observed for 27 of the 45 responses; for the remaining 18 responses, ratings differed by no more than one adjacent CUS category. Detailed reliability results are presented in Supplementary Table S2.

### 3.2. Platform Differences in Expert-Rated Outcomes

At the response level, after averaging the three pediatrician ratings for each vignette–platform combination, mean (SD) mDISCERN total scores were 20.36 (5.21) for ChatGPT, 22.11 (4.25) for Gemini, and 22.36 (3.71) for Grok. The corresponding mean (SD) CUS scores were 4.07 (1.17), 4.47 (1.00), and 4.51 (0.84), respectively.

Mean CUS scores did not differ significantly across the three platforms (Friedman χ^2^(2) = 3.707, *p* = 0.157). Mean mDISCERN scores differed across platforms in the global Friedman test (χ^2^(2) = 6.533, *p* = 0.038). However, none of the pairwise comparisons remained statistically significant after Bonferroni adjustment: ChatGPT versus Gemini (adjusted *p* = 0.452), ChatGPT versus Grok (adjusted *p* = 0.460), and Gemini versus Grok (adjusted *p* = 1.000). Detailed global and pairwise comparisons are presented in Supplementary Table S1.

### 3.3. Diagnostic Correctness and Vignette-Level Variation

In this single-query descriptive assessment, the documented reference diagnosis was identified as the most likely diagnosis in 9 of 15 vignettes (60.0%) by ChatGPT, 12 of 15 (80.0%) by Gemini, and 13 of 15 (86.7%) by Grok. At least one platform failed to identify the reference diagnosis in 9 of the 15 vignettes. These proportions represent one generated response for each vignette–platform combination and were not subjected to inferential comparison.

ChatGPT did not identify the reference diagnosis in vignettes 1 (erythema ab igne), 7 (acrodermatitis enteropathica), 8 (pustular psoriasis), 9 (variegate porphyria), 11 (papular acrodermatitis of childhood), and 15 (infectious mononucleosis). Gemini did not identify the reference diagnosis in vignettes 4 (ecthyma gangrenosum), 9 (variegate porphyria), and 13 (bullous impetigo), whereas Grok did not identify the reference diagnosis in vignettes 3 (insect bite) and 9 (variegate porphyria). All three platforms failed to identify the reference diagnosis in vignette 9. Vignette-level diagnostic classifications are presented in Supplementary Table S3.

Expert ratings also varied substantially across vignettes. Across the nine ratings available for each vignette, pooled mean CUS scores ranged from 2.00 to 5.00, whereas pooled mean mDISCERN totals ranged from 10.00 to 23.89. Vignette 9, representing porphyria, received the lowest scores from all three platforms, with identical platform means for both CUS (2.00) and mDISCERN (10.00).

The largest between-platform differences in mean CUS scores were observed for vignettes 4, 7, 11, and 15, each with a range of 2.67 points. The largest between-platform difference in mean mDISCERN scores occurred for vignette 7 (range, 13.33), followed by vignettes 11 (10.33), 4 (10.00), and 15 (9.33). Gemini received the lowest ratings for vignette 4, whereas ChatGPT received the lowest ratings for vignettes 7, 11, and 15. Complete vignette-level expert-rating results are presented in Supplementary Table S4.

Illustrative discordant outputs included prioritization of a drug eruption rather than the underlying infectious mononucleosis diagnosis, identification of scabies in the insect-bite vignette, and failure of all three platforms to identify porphyria. The corresponding redacted clinical photographs, full vignette texts, and complete model-generated responses are provided in Supplementary File S1.

### 3.4. Response Length and Readability

Response length and readability differed across platforms (Table 3). Gemini generated the longest responses, with a mean (SD) word count of 757.7 (67.2), compared with 543.3 (74.5) for ChatGPT and 518.4 (106.1) for Grok, whereas Grok produced the lowest Flesch Reading Ease scores and the highest grade-level estimates. Significant platform differences were observed for word count, sentence count, and all five readability indices (all *p* < 0.001). Full adjusted pairwise comparisons are provided in Supplementary Table S5.

Despite these linguistic differences, associations with expert-rated clinical outcomes were uniformly weak and non-significant. Across the 45 vignette–platform responses, |ρ| was ≤0.063 for mean CUS and ≤0.097 for mean mDISCERN, and none of the 14 exploratory correlations remained significant after Benjamini–Hochberg adjustment. Full correlation results are presented in Supplementary Table S6, and vignette-level response-length and readability measures are presented in Supplementary Table S7.

## 4. Discussion

### 4.1. Principal Findings and Comparison with Previous Research

In this comparative vignette-based study, three widely accessible multimodal large language models generated predominantly clinically acceptable responses to pediatric rash presentations. Overall, 82.2% of Clinical Utility and Safety (CUS) ratings were in categories 4–5, 83.0% of modified DISCERN (mDISCERN) scores were at least 20 out of 25, and no rating was assigned to CUS category 1. Gemini and Grok had descriptively higher mean CUS and mDISCERN scores than ChatGPT. However, CUS scores did not differ significantly across platforms in the vignette-level comparison. Although the global comparison of mDISCERN scores was statistically significant, none of the individual pairwise differences remained significant after adjustment for multiple comparisons. These findings suggest potential platform-level variation but do not provide definitive evidence that one model consistently outperformed the others across the evaluated vignettes. Importantly, the absence of category 1 ratings should not be interpreted as evidence that the responses were free of safety-relevant errors, because diagnostically incorrect or substantially incomplete responses could still receive higher CUS categories.

The generally favorable expert ratings are consistent with multidimensional evaluations of LLM-generated health information in other clinical areas. Studies addressing gestational diabetes and hypothyroidism during pregnancy have reported predominantly acceptable reliability and readability, while also identifying variation in completeness and quality between platforms [25,26]. Similarly, a multidimensional evaluation of LLM-generated guidance for laparoscopic cholecystectomy found that responses could provide useful clinical information but still differed in reliability, completeness, and linguistic complexity [27]. Although these studies addressed different populations and clinical tasks, their overall findings resemble the pattern observed here: contemporary LLMs frequently produce clinically plausible and informative responses, but output quality remains dependent on both the platform and the clinical task.

An important distinction is that the present study evaluated clinician-facing, open-ended outputs rather than answers to isolated factual questions or patient information requests. Each response included a most likely diagnosis, ranked differential diagnoses, immediate management recommendations, and safety considerations. Consequently, the CUS and mDISCERN ratings reflected the overall clinical value of the generated response rather than diagnostic correctness alone. This broader evaluation is relevant because an accurate diagnostic label may still be accompanied by incomplete management advice, insufficient acknowledgment of uncertainty, or inadequate safety guidance. Conversely, a response that does not identify the documented diagnosis may still contain useful differential diagnoses or appropriate recommendations for further assessment.

### 4.2. Diagnostic Performance and Vignette-Level Interpretation

In this single-query descriptive assessment, the documented reference diagnosis was identified as the most likely diagnosis in 60.0% of vignettes by ChatGPT, 80.0% by Gemini, and 86.7% by Grok. Because each vignette–platform combination generated only one response, these proportions represent observed single-response outcomes rather than stable estimates of platform-level diagnostic accuracy or a reproducible ranking of the three models. Nevertheless, they demonstrate meaningful variation in diagnostic performance, with at least one platform failing to identify the reference diagnosis in 9 of the 15 vignettes.

Studies conducted in pediatric emergency settings have reported encouraging but inconsistent LLM diagnostic performance, with accuracy varying according to the model, clinical presentation, and amount of contextual information supplied [7,8]. More recently, a large study evaluated ChatGPT, Gemini, and Copilot against 263 pediatric residents and specialists using 61 image-supported, multiple-choice childhood exanthem cases. ChatGPT and Gemini achieved scores above the upper bound of the specialist population median, but disease-level accuracy varied substantially across conditions [14]. Together, these findings indicate that favorable aggregate performance does not guarantee reliable recognition of every pediatric rash presentation.

The present study complements the previous diagnostic-accuracy literature by assessing open-ended clinical reasoning. The recent 61-case study primarily examined whether a model selected the correct diagnosis from predefined alternatives [14]. In contrast, the models in the present study independently generated a primary diagnosis, differential diagnoses, management recommendations, and safety warnings. The current findings therefore provide additional information about the clinical quality of the reasoning and guidance accompanying the diagnostic label.

Performance also differed considerably by vignette. Vignette 9, representing variegate porphyria, received the lowest CUS and mDISCERN scores, and none of the three platforms identified the documented reference diagnosis. The reference diagnosis of variegate porphyria was established based on the compatible clinical presentation, supportive biochemical findings, and clinical response to hemin therapy. All three platforms received identical mean scores for this vignette, indicating a shared failure rather than an isolated platform-specific error. Similar case-dependent limitations have been reported in broader evaluations of clinical LLMs, particularly when successful interpretation requires integration of subtle contextual information rather than recognition of a familiar textbook pattern [9,14]. This shared difficulty suggests that uncommon or clinically ambiguous presentations remain challenging for general-purpose multimodal systems. However, without a clinician comparator completing the same open-ended task, this shared model failure cannot be interpreted as evidence that human clinicians would necessarily have identified the diagnosis from the same limited vignette information.

Marked between-platform variation was also observed in vignettes 4, 7, 11, and 15. Gemini received substantially lower ratings for the ecthyma gangrenosum vignette, whereas ChatGPT received the lowest ratings for the acrodermatitis enteropathica, papular acrodermatitis of childhood, and infectious mononucleosis vignettes. The largest difference in mDISCERN scores occurred in the acrodermatitis enteropathica vignette. These findings should not be attributed to specific cognitive mechanisms such as anchoring or premature closure, which cannot be directly inferred from model-generated text. They instead illustrate that performance may vary considerably between clinical presentations and that errors can occur in both uncommon and relatively recognizable conditions. Illustrative errors included prioritizing a drug eruption rather than the underlying infectious mononucleosis diagnosis, identifying the insect-bite vignette as scabies, and failure of all three platforms to recognize porphyria.

This variability has practical implications. Aggregate diagnostic percentages and mean quality scores may obscure isolated but clinically important failures. General-purpose LLMs may help broaden a differential diagnosis or organize an initial approach, but their recommendations still require verification against the clinical examination, disease evolution, laboratory findings, and established pediatric guidance. The findings therefore support supervised use rather than reliance on these systems as autonomous diagnostic tools.

### 4.3. Response Length, Readability, and Clinical Quality

The platforms also differed substantially in response length and readability. Gemini generated the longest responses, whereas Grok produced the lowest Flesch Reading Ease scores and the highest grade-level estimates. Despite these differences, response length and readability were not meaningfully associated with expert-rated clinical utility, safety, or information quality.

Previous studies have similarly shown that LLM-generated medical information frequently exceeds conventional readability targets. Evaluations in pregnancy-related endocrinology, surgical guidance, and orthopedic patient information have reported technically complex or high-grade-level outputs despite generally acceptable information quality [25,26,27,28]. These findings suggest that readability and clinical quality represent related but distinct dimensions of LLM performance.

The lack of a meaningful association in the present study may partly reflect the intended audience. The prompts were designed for pediatric residents and requested differential diagnoses, immediate management, and safety considerations. Traditional readability formulas primarily quantify sentence length, syllable count, word length, or character density; they do not directly assess clinical reasoning, diagnostic accuracy, prioritization, or the practical relevance of a recommendation. A technically complex response may therefore receive a high expert rating when it is accurate, well organized, and clinically actionable.

Accordingly, greater response length or linguistic complexity should not be interpreted as evidence of higher clinical quality. In time-sensitive settings, concise and clearly structured outputs may be easier to review, although this was not directly evaluated in the present study. Our study also did not measure reading time, comprehension, or clinician workload. Future studies should therefore evaluate whether concise and structured LLM responses improve the efficiency with which clinicians recognize diagnoses, warning signs, and management priorities.

### 4.4. Clinical and Educational Implications

The evaluation of general-purpose rather than dermatology-specific systems was a deliberate feature of this study. Specialized image classifiers and dermatology-focused multimodal models may achieve strong performance within defined tasks [11,12,13]. However, pediatricians working in outpatient clinics, emergency departments, or general wards are more likely to access widely available conversational platforms than a dedicated dermatologic AI system. The present findings therefore reflect the performance of tools that clinicians and trainees may realistically encounter in routine practice. The potential role of these systems may also differ according to specialty. Pediatricians typically interpret skin findings together with age, systemic symptoms, infectious exposures, growth and development, and laboratory data, whereas dermatologists bring greater expertise in subtle lesion morphology, distribution, and dermatologic differential diagnosis. Accordingly, multimodal LLM outputs may have different strengths and limitations depending on the expertise of the clinician using them. Because the present responses were evaluated by pediatricians rather than dermatologists, our findings should not be extrapolated to specialist dermatologic assessment.

The results suggest several potential supervised uses. An LLM response may serve as a diagnostic checklist, prompt consideration of alternative diagnoses, organize an initial management plan, or highlight the need for dermatology consultation. These functions may be particularly valuable when a trainee encounters an unfamiliar rash or when the differential diagnosis is broad. However, the output should be treated as a starting point for clinical review rather than an authoritative recommendation. In practice, such outputs may be most appropriately used to broaden or structure a differential diagnosis, while the proposed primary diagnosis, management recommendations, and safety statements are independently verified against the clinical examination, disease course, laboratory findings, and established guidance.

A related educational application is the use of LLM responses as material for critical appraisal. Structured evaluations in anatomical education have suggested that AI-generated explanations can support discussion and learning when their accuracy, limitations, and ethical implications are explicitly examined [29,30]. In pediatric training, comparing an LLM response with the documented diagnosis and expert reasoning could help residents identify omitted differential diagnoses, assess whether safety warnings are adequate, and recognize potentially misleading confidence. The present study did not directly measure educational outcomes, and this proposed role should therefore be evaluated prospectively.

### 4.5. Strengths and Limitations

This study has several strengths, including the use of real pediatric vignettes with clinical photographs, standardized multimodal prompting across three widely accessible platforms, and independent assessment by three pediatricians blinded to platform identity. The evaluation extended beyond diagnostic correctness to include clinical utility, safety, information quality, response length, and readability. Complete vignette materials, redacted photographs, and model-generated responses are provided in the Supplementary Materials, allowing direct inspection of the evaluated outputs.

Several limitations should be considered. First, the study included a small, purposively selected set of 15 vignettes from a single tertiary pediatric center, and five initially considered vignettes were excluded because they were considered overly straightforward. The retained set was therefore enriched for diagnostically challenging presentations, and the observed diagnostic correctness proportions should not be interpreted as prevalence-weighted estimates of real-world performance. Each vignette was submitted only once to each platform, so the diagnostic findings represent single-response observations and may be affected by run-to-run variability. In addition, no clinician comparator group completed the same open-ended task; therefore, the study cannot determine whether the evaluated LLMs performed better or worse than pediatricians or dermatologists on these vignettes.

The expert raters were blinded to platform identity but were familiar with the source patients and aware of the documented reference diagnoses; consequently, CUS and mDISCERN ratings were reference-informed and may have been influenced by diagnostic concordance. CUS was study-specific and mDISCERN was adapted for clinician-facing responses, and neither instrument has been externally validated for this setting. The high mDISCERN ICC estimates should also be interpreted in light of the substantial between-response variability, which can influence ICC magnitude. Furthermore, the models evaluated static photographs and selected clinical information rather than complete bedside examinations, and all prompts and outputs were in English; both LLM performance and readability may differ in other languages [31]. Finally, the study evaluated model outputs rather than the effect of LLM assistance on clinician performance or patient outcomes.

## 5. Conclusions

Widely accessible multimodal LLMs generated predominantly clinically acceptable responses to pediatric rash vignettes, but their performance varied across clinical presentations. No consistent platform advantage was demonstrated after adjustment for pairwise comparisons, and the single-query diagnostic findings should be interpreted as observations from the sampled responses rather than stable estimates of platform accuracy. All three platforms failed to identify the documented diagnosis in at least one challenging vignette, underscoring the importance of clinical verification.

These findings support further evaluation of LLMs as supervised adjunctive tools for differential diagnosis, clinical review, and pediatric education rather than as autonomous diagnostic systems. Larger, prospectively designed studies should assess repeated outputs, clinician comparator groups, multilingual performance, more diverse clinical presentations, and the direct effect of LLM assistance on clinician performance and patient care.

## Figures and Tables

**Table 1:** Quality, Safety, and Readability Measures Used to Evaluate LLM Responses to Pediatric Rash Vignettes.

Metric	Operational Content	Interpretation
**CUS (Clinical Utility and Safety)**	Study-specific single-item anchored rating of the overall clinical usefulness and safety of the LLM response: 1. Clinically dangerous or clearly incorrect advice; 2. Markedly incorrect or substantially incomplete advice; 3. Partly correct response, but important clinical direction or safety information is missing; 4. Generally correct and safe, with only minor omissions or ambiguities; 5. Fully correct, safe, and broadly consistent with current pediatric practice.	Direct score from 1 to 5. Higher scores indicate greater clinical usefulness, safety, and concordance with pediatric guidance.
**mDISCERN (modified DISCERN)**	Five-item instrument adapted for clinician-facing guidance: 1. Scientific grounding and use of evidence; 2. Balance and acknowledgment of alternatives or uncertainty; 3. Clarity regarding risks, benefits, and contraindications; 4. Concordance with current pediatric standards; 5. Overall reliability, clarity, and suitability for the clinical context.	Each item is scored from 1 to 5; total score range 5–25. Higher scores indicate more reliable, complete, balanced, and clinically appropriate information.
**FRES (Flesch Reading Ease Score)**	Readability index based primarily on sentence length and word complexity.	Higher scores indicate easier readability. No adequacy threshold was prespecified because the responses were clinician-facing.
**FKGL (Flesch–Kincaid Grade Level)**	Grade-level estimate of text difficulty based on sentence length and syllable count.	Expressed as an approximate US educational grade level; higher values indicate more complex text.
**GFI (Gunning Fog Index)**	Readability estimate incorporating sentence length and the proportion of complex words.	Expressed as an approximate educational grade level; higher values indicate greater reading difficulty.
**SMOG (Simple Measure of Gobbledygook)**	Readability estimate based primarily on the frequency of polysyllabic words.	Estimates the years of education required to understand the text; higher scores indicate greater linguistic complexity.
**CLI (Coleman–Liau Index)**	Readability estimate based on the number of letters and sentences per 100 words.	Expressed as an approximate US educational grade level; higher values indicate more complex or technically dense text.
**Word and sentence counts**	Total numbers of words and sentences in each response.	Indicators of response length only; higher values indicate longer responses but do not necessarily indicate greater quality or completeness.

Note: CUS was developed specifically for the present study. The mDISCERN instrument was adapted from the original DISCERN framework for the evaluation of clinician-facing LLM responses.

**Table 2:** Expert-Rated Clinical Quality and Safety by LLM Platform.

Platform	CUS, Mean (SD) ^a^	CUS Categories 4–5, *n* (%) ^b^	mDISCERN Total, Mean (SD) ^a^	mDISCERN ≥20, *n* (%) ^b^
ChatGPT	4.07 (1.17)	33 (73.3)	20.36 (5.21)	33 (73.3)
Gemini	4.47 (1.00)	38 (84.4)	22.11 (4.25)	39 (86.7)
Grok	4.51 (0.84)	40 (88.9)	22.36 (3.71)	40 (88.9)

Note: ^a^ Means and standard deviations were calculated from the 15 vignette–platform response-level means per platform after averaging the three rater scores for each response. ^b^ Categorical proportions were calculated from the 45 individual ratings per platform. CUS categories 4–5 indicate responses rated as generally or fully correct, clinically useful, and safe. An mDISCERN total score ≥ 20 corresponds to a mean item score of at least 4/5 and was used descriptively to indicate high information quality. CUS, Clinical Utility and Safety; mDISCERN, modified DISCERN.

**Table 3:** Response Length and Readability Measures by LLM Platform.

Platform	Word Count, Mean (SD)	Sentence Count, Mean (SD)	FRES, Mean (SD)	FKGL, Mean (SD)	GFI, Mean (SD)	CLI, Mean (SD)	SMOG, Mean (SD)
ChatGPT	543.3 (74.5)	36.9 (7.3)	22.63 (8.89)	13.90 (1.37)	15.29 (1.93)	15.81 (1.39)	14.81 (1.49)
Gemini	757.7 (67.2)	44.3 (3.0)	19.97 (8.88)	14.71 (1.33)	15.78 (1.33)	15.94 (1.45)	15.79 (0.95)
Grok	518.4 (106.1)	31.0 (7.9)	8.49 (10.03)	15.67 (1.55)	17.49 (1.71)	18.38 (1.55)	16.87 (1.82)

Note: Each platform contributed 15 responses. FRES, Flesch Reading Ease Score; FKGL, Flesch–Kincaid Grade Level; GFI, Gunning Fog Index; CLI, Coleman–Liau Index; SMOG, Simple Measure of Gobbledygook.

## Data Availability

The data supporting the findings of this study are available with the article. The Supplementary Materials include the full vignette texts, redacted clinical photographs, and LLM-generated responses, and the de-identified numerical dataset used for the analyses has been uploaded separately as an associated data file. Original, unredacted clinical photographs are not publicly available because of privacy and ethical restrictions.

## Supplementary Materials

The following supporting information can be downloaded at https://www.mdpi.com/article/10.3390/children13091272/s1. Supplementary Table S1: Vignette-Level Platform Comparisons for Expert-Rated Outcomes; Supplementary Table S2: Inter-Rater Reliability for Expert-Rated Outcomes; Supplementary Table S3: Vignette-Level Reference Diagnoses and Diagnostic Correctness by Platform; Supplementary Table S4: Vignette-Level Expert Score Summaries and Cross-Platform Variation; Supplementary Table S5: Pairwise Platform Comparisons for Response Length and Readability Measures; Supplementary Table S6: Exploratory Associations Between Expert-Rated Outcomes and Response Length or Readability; Supplementary Table S7: Vignette-Level Variation in Response Length and Readability across Platforms; Supplementary File S1: Full vignette texts, redacted clinical photographs, and LLM-generated responses.
