# Supplementary material for: Comparative Expert Evaluation of Multimodal Large Language Models for Pediatric Rash Diagnosis: Clinical Utility, Safety, Information Quality, and Readability
Source: Children (Basel). 2026 Sep 18;13(9):1272. doi: 10.3390/children13091272 (PMC13605588; doi:10.3390/children13091272)
Supplement: Supplementary file 1 [file children-13-01272-s001.zip › Supplementary Tables.pdf]

Supplementary Tables

Supplementary Table S1. Vignette-Level Platform Comparisons for Expert-Rated Outcomes

Panel A. Overall Friedman Tests

Outcome	Friedman chi-square	df	p Value
CUS	3.707	2	0.157
mDISCERN total	6.533	2	0.038

Panel B. Pairwise Wilcoxon Signed-Rank Tests for mDISCERN Total Scores

Comparison	V Statistic	Raw p Value	Bonferroni-Adjusted p Value
ChatGPT vs. Gemini	24.5	0.151	0.452
ChatGPT vs. Grok	13.0	0.153	0.460
Gemini vs. Grok	22.0	0.624	1.000

Note: Ratings were averaged across the three raters for each vignette-platform combination, yielding 15 matched observations per platform. Pairwise Wilcoxon signed-rank tests were performed only for mDISCERN because the global Friedman test was statistically significant. Asymptotic two-sided p values with continuity correction were used because tied differences were present.

Supplementary Table S2. Inter-Rater Reliability for Expert-Rated Outcomes

Outcome	Reliability Statistic	Estimate	95% Confidence Interval
mDISCERN total	ICC(2,1), single rater	0.964	0.938-0.980
mDISCERN total	ICC(2,3), mean of three raters	0.988	0.979-0.993
CUS	Krippendorff's alpha, ordinal	0.743	Not calculated

Note: ICC estimates were calculated across the 45 vignette-platform responses using a two-way random-effects model for absolute agreement. ICC(2,1) represents the reliability of a single rater, and ICC(2,3) represents the reliability of the mean of three raters. CUS agreement was calculated using Krippendorff's alpha with an ordinal distance metric. All three raters assigned identical CUS scores to 27 of 45 responses; ratings for the remaining 18 responses differed by no more than one adjacent category.

Supplementary Table S3. Vignette-Level Reference Diagnoses and Diagnostic Correctness by Platform

Vignette ID	Reference Diagnosis	ChatGPT	Gemini	Grok
1	Erythema ab igne	Incorrect	Correct	Correct
2	Eczema herpeticum	Correct	Correct	Correct
3	Insect bite	Correct	Correct	Incorrect
4	Ecthyma gangrenosum	Correct	Incorrect	Correct
5	IgA vasculitis (Henoch-Schönlein purpura)	Correct	Correct	Correct
6	Herpes zoster	Correct	Correct	Correct
7	Acrodermatitis enteropathica	Incorrect	Correct	Correct
8	Pustular psoriasis	Incorrect	Correct	Correct
9	Variegate Porphyria	Incorrect	Incorrect	Incorrect
10	Juvenile dermatomyositis	Correct	Correct	Correct
11	Papular acrodermatitis of childhood	Incorrect	Correct	Correct
12	Café-au-lait macules	Correct	Correct	Correct
13	Bullous impetigo	Correct	Incorrect	Correct
14	Roseola infantum	Correct	Correct	Correct
15	Infectious mononucleosis	Incorrect	Correct	Correct
Summary	Correct, n (%)	9/15 (60.0%)	12/15 (80.0%)	13/15 (86.7%)

Note: A response was classified as correct only when the diagnosis stated as the most likely diagnosis matched the documented reference diagnosis or represented an unequivocal synonym. Broader or nonspecific categories, clinically related but distinct conditions, and reference diagnoses mentioned only within the differential diagnosis were classified as incorrect. Reference diagnosis of variegate porphyria was supported by elevated urinary porphobilinogen during the diagnostic work-up. Genetic testing identified a variant of uncertain significance and was not considered independently confirmatory.

Supplementary Table S4. Vignette-Level Expert Score Summaries and Cross-Platform Variation

Panel A. Clinical Utility and Safety Scores

ID	Reference Diagnosis	Pooled Mean (SD)	ChatGPT Mean	Gemini Mean	Grok Mean	Range	Lowest Platform(s)	Highest Platform(s)
1	Erythema ab igne	4.89 (0.33)	4.67	5.00	5.00	0.33	ChatGPT	Gemini/Grok
2	Eczema herpeticum	4.67 (0.50)	4.67	4.67	4.67	0.00	All	All
3	Insect bite	4.67 (0.50)	5.00	5.00	4.00	1.00	Grok	ChatGPT/Gemini
4	Ecthyma gangrenosum	4.11 (1.36)	5.00	2.33	5.00	2.67	Gemini	ChatGPT/Grok
5	IgA vasculitis (Henoch-Schönlein purpura)	4.44 (0.53)	4.00	5.00	4.33	1.00	ChatGPT	Gemini
6	Herpes zoster	4.89 (0.33)	4.67	5.00	5.00	0.33	ChatGPT	Gemini/Grok
7	Acrodermatitis enteropathica	4.00 (1.32)	2.33	4.67	5.00	2.67	ChatGPT	Grok
8	Pustular psoriasis	4.78 (0.44)	4.33	5.00	5.00	0.67	ChatGPT	Gemini/Grok
9	Variegate Porphyria	2.00 (0.00)	2.00	2.00	2.00	0.00	All	All
10	Juvenile dermatomyositis	5.00 (0.00)	5.00	5.00	5.00	0.00	All	All
11	Papular acrodermatitis of childhood	4.00 (1.32)	2.33	5.00	4.67	2.67	ChatGPT	Gemini
12	Café-au-lait macules	4.89 (0.33)	4.67	5.00	5.00	0.33	ChatGPT	Gemini/Grok
13	Bullous impetigo	4.44 (0.73)	5.00	3.67	4.67	1.33	Gemini	ChatGPT
14	Roseola infantum	4.33 (0.87)	5.00	4.67	3.33	1.67	Grok	ChatGPT
15	Infectious mononucleosis	4.11 (1.36)	2.33	5.00	5.00	2.67	ChatGPT	Gemini/Grok

Note: Pooled means and standard deviations were calculated across the nine CUS ratings available for each vignette (three platforms x three raters). Platform means were calculated across the three raters. Range is the difference between the highest and lowest platform mean; tied platforms are listed together.

Supplementary Table S4. Continued

Panel B. Modified DISCERN Total Scores

ID	Reference Diagnosis	Pooled Mean (SD)	ChatGPT Mean	Gemini Mean	Grok Mean	Range	Lowest Platform(s)	Highest Platform(s)
1	Erythema ab igne	23.67 (0.71)	23.00	24.00	24.00	1.00	ChatGPT	Gemini/Grok
2	Eczema herpeticum	23.00 (0.87)	22.00	23.67	23.33	1.67	ChatGPT	Gemini
3	Insect bite	23.22 (1.20)	24.00	24.00	21.67	2.33	Grok	ChatGPT/Gemini
4	Ecthyma gangrenosum	20.67 (5.07)	24.00	14.00	24.00	10.00	Gemini	ChatGPT/Grok
5	IgA vasculitis (Henoch-Schönlein purpura)	22.89 (1.36)	22.33	24.00	22.33	1.67	ChatGPT/Grok	Gemini
6	Herpes zoster	23.78 (0.44)	23.67	24.00	23.67	0.33	ChatGPT/Grok	Gemini
7	Acrodermatitis enteropathica	19.56 (6.67)	10.67	24.00	24.00	13.33	ChatGPT	Gemini/Grok
8	Pustular psoriasis	23.44 (1.13)	22.33	24.00	24.00	1.67	ChatGPT	Gemini/Grok
9	Variegate Porphyria	10.00 (0.00)	10.00	10.00	10.00	0.00	All	All
10	Juvenile dermatomyositis	23.78 (0.44)	23.67	24.00	23.67	0.33	ChatGPT/Grok	Gemini
11	Papular acrodermatitis of childhood	20.56 (5.36)	13.67	24.00	24.00	10.33	ChatGPT	Gemini/Grok
12	Café-au-lait macules	23.89 (0.33)	23.67	24.00	24.00	0.33	ChatGPT	Gemini/Grok
13	Bullous impetigo	22.89 (1.54)	23.67	21.00	24.00	3.00	Gemini	Grok
14	Roseola infantum	21.89 (2.67)	24.00	23.00	18.67	5.33	Grok	ChatGPT
15	Infectious mononucleosis	20.89 (4.78)	14.67	24.00	24.00	9.33	ChatGPT	Gemini/Grok

Note: Pooled means and standard deviations were calculated across the nine mDISCERN ratings available for each vignette (three platforms x three raters). Platform means were calculated across the three raters. Range is the difference between the highest and lowest platform mean; tied platforms are listed together.

Supplementary Table S5. Pairwise Platform Comparisons for Response Length and Readability Measures

Outcome	Comparison	Mean Difference	SE	df	t	Raw p Value	BH-Adjusted p Value
Word count	ChatGPT vs. Gemini	-214.33	23.88	14	-8.975	<0.001	<0.001
Word count	ChatGPT vs. Grok	24.93	27.40	14	0.910	0.378	0.378
Word count	Gemini vs. Grok	239.27	22.26	14	10.749	<0.001	<0.001
Sentence count	ChatGPT vs. Gemini	-7.47	1.96	14	-3.812	0.002	0.003
Sentence count	ChatGPT vs. Grok	5.87	2.68	14	2.185	0.046	0.046
Sentence count	Gemini vs. Grok	13.33	2.07	14	6.436	<0.001	<0.001
FRES	ChatGPT vs. Gemini	2.67	1.87	14	1.425	0.176	0.176
FRES	ChatGPT vs. Grok	14.15	1.73	14	8.155	<0.001	<0.001
FRES	Gemini vs. Grok	11.48	1.66	14	6.911	<0.001	<0.001
FKGL	ChatGPT vs. Gemini	-0.81	0.35	14	-2.322	0.036	0.036
FKGL	ChatGPT vs. Grok	-1.77	0.35	14	-5.002	<0.001	<0.001
FKGL	Gemini vs. Grok	-0.96	0.29	14	-3.330	0.005	0.007
GFI	ChatGPT vs. Gemini	-0.49	0.47	14	-1.027	0.322	0.322
GFI	ChatGPT vs. Grok	-2.19	0.48	14	-4.606	<0.001	<0.001
GFI	Gemini vs. Grok	-1.71	0.32	14	-5.257	<0.001	<0.001
CLI	ChatGPT vs. Gemini	-0.13	0.28	14	-0.448	0.661	0.661
CLI	ChatGPT vs. Grok	-2.57	0.35	14	-7.378	<0.001	<0.001
CLI	Gemini vs. Grok	-2.44	0.29	14	-8.340	<0.001	<0.001
SMOG	ChatGPT vs. Gemini	-0.98	0.39	14	-2.503	0.025	0.030
SMOG	ChatGPT vs. Grok	-2.05	0.56	14	-3.678	0.002	0.007
SMOG	Gemini vs. Grok	-1.07	0.44	14	-2.423	0.030	0.030

Note: Comparisons used paired-samples t tests across the 15 matched vignettes. Mean differences are shown as the first platform minus the second platform. Benjamini-Hochberg adjustment was applied separately within each outcome across its three pairwise comparisons. BH, Benjamini-Hochberg; SE, standard error.

Supplementary Table S6. Exploratory Associations between Expert-Rated Outcomes and Response Length or Readability

Expert-Rated Outcome	Text Metric	Spearman rho	Raw p Value	BH-Adjusted p Value	n
Mean CUS	Word count	-0.035	0.818	0.963	45
Mean CUS	Sentence count	-0.054	0.726	0.963	45
Mean CUS	FRES	0.050	0.743	0.963	45
Mean CUS	FKGL	-0.063	0.682	0.963	45
Mean CUS	GFI	-0.062	0.685	0.963	45
Mean CUS	CLI	-0.029	0.850	0.963	45
Mean CUS	SMOG	0.026	0.866	0.963	45
Mean mDISCERN	Word count	0.010	0.947	0.963	45
Mean mDISCERN	Sentence count	-0.063	0.680	0.963	45
Mean mDISCERN	FRES	0.015	0.922	0.963	45
Mean mDISCERN	FKGL	-0.009	0.951	0.963	45
Mean mDISCERN	GFI	0.012	0.938	0.963	45
Mean mDISCERN	CLI	-0.007	0.963	0.963	45
Mean mDISCERN	SMOG	0.097	0.526	0.963	45

Note: Spearman correlations were calculated at the vignette-platform level using CUS and mDISCERN ratings averaged across the three raters (n = 45). Benjamini-Hochberg adjustment was applied across all 14 exploratory correlations. FRES, Flesch Reading Ease Score; FKGL, Flesch-Kincaid Grade Level; GFI, Gunning Fog Index; CLI, Coleman-Liau Index; SMOG, Simple Measure of Gobbledygook.

Supplementary Table S7. Vignette-Level Variation in Response Length and Readability across Platforms

Panel A. Response Length

ID	Reference Diagnosis	Word Count Mean	Word Count Range	Sentence Count Mean	Sentence Count Range
1	Erythema ab igne	612.3	275	32.7	22.0
2	Eczema herpeticum	603.7	337	37.0	15.0
3	Insect bite	519.7	282	38.7	11.0
4	Ecthyma gangrenosum	628.0	443	42.3	20.0
5	IgA vasculitis (Henoch-Schönlein purpura)	566.0	335	41.7	13.0
6	Herpes zoster	580.7	407	36.7	26.0
7	Acrodermatitis enteropathica	676.7	246	38.7	11.0
8	Pustular psoriasis	641.7	320	36.3	19.0
9	Variegate Porphyria	779.7	144	44.3	11.0
10	Juvenile dermatomyositis	634.0	177	41.7	16.0
11	Papular acrodermatitis of childhood	566.0	190	33.0	19.0
12	Café-au-lait macules	530.0	352	29.7	24.0
13	Bullous impetigo	564.7	174	37.0	13.0
14	Roseola infantum	582.3	153	34.3	9.0
15	Infectious mononucleosis	611.7	196	37.0	17.0

Note: For each vignette, mean values were calculated across the three platform responses. Range is the difference between the maximum and minimum platform values.

Supplementary Table S7. Continued

Panel B. FRES, FKGL, and GFI

ID	Reference Diagnosis	FRES Mean	FRES Range	FKGL Mean	FKGL Range	GFI Mean	GFI Range
1	Erythema ab igne	23.3	9.3	14.3	0.8	16.4	2.2
2	Eczema herpeticum	9.8	13.6	15.9	0.3	18.0	0.9
3	Insect bite	25.3	4.2	13.1	2.5	14.0	3.9
4	Ecthyma gangrenosum	8.3	13.7	15.7	4.1	17.1	4.2
5	IgA vasculitis (Henoch-Schönlein purpura)	15.7	10.5	14.5	0.9	15.9	2.2
6	Herpes zoster	18.2	17.4	14.5	2.1	15.6	2.6
7	Acrodermatitis enteropathica	9.0	22.1	16.0	2.9	17.7	3.6
8	Pustular psoriasis	7.3	16.5	16.4	2.2	18.4	3.3
9	Variegate Porphyria	8.8	24.7	16.1	2.0	17.6	2.6
10	Juvenile dermatomyositis	12.8	18.4	15.1	3.4	15.8	4.6
11	Papular acrodermatitis of childhood	15.4	25.8	14.8	2.9	16.1	3.5
12	Café-au-lait macules	30.1	19.2	13.3	2.5	14.9	3.3
13	Bullous impetigo	31.6	10.4	12.6	2.1	14.1	2.8
14	Roseola infantum	27.2	16.8	13.5	0.8	14.9	1.4
15	Infectious mononucleosis	12.8	14.5	15.5	2.9	16.3	3.3

Note: For each vignette, mean values were calculated across the three platform responses. Range is the difference between the maximum and minimum platform values. FRES, Flesch Reading Ease Score; FKGL, Flesch-Kincaid Grade Level; GFI, Gunning Fog Index.

Supplementary Table S7. Continued

Panel C. CLI and SMOG

ID	Reference Diagnosis	CLI Mean	CLI Range	SMOG Mean	SMOG Range
1	Erythema ab igne	15.7	2.8	16.4	2.8
2	Eczema herpeticum	17.4	2.4	16.7	1.5
3	Insect bite	16.3	0.7	13.9	3.4
4	Ecthyma gangrenosum	18.0	2.2	16.1	5.9
5	IgA vasculitis (Henoch-Schönlein purpura)	16.6	1.6	14.9	3.0
6	Herpes zoster	16.5	3.2	15.5	2.4
7	Acrodermatitis enteropathica	17.7	3.7	17.2	3.2
8	Pustular psoriasis	18.4	3.2	17.3	2.6
9	Variegate Porphyria	18.4	4.9	16.8	1.4
10	Juvenile dermatomyositis	17.8	2.5	15.7	3.9
11	Papular acrodermatitis of childhood	16.7	5.0	16.4	4.3
12	Café-au-lait macules	14.8	3.9	15.2	2.6
13	Bullous impetigo	14.4	1.9	14.3	1.9
14	Roseola infantum	15.0	3.8	15.0	0.6
15	Infectious mononucleosis	17.0	2.5	16.0	2.8

Note: For each vignette, mean values were calculated across the three platform responses. Range is the difference between the maximum and minimum platform values. CLI, Coleman-Liau Index; SMOG, Simple Measure of Gobbledygook.
